# Endoscopic Management of Weight Recurrence Following Bariatric Surgery

**DOI:** 10.3389/fendo.2022.946870

**Published:** 2022-07-14

**Authors:** Donna Maria Abboud, Rebecca Yao, Babusai Rapaka, Rabih Ghazi, Omar M. Ghanem, Barham K. Abu Dayyeh

**Affiliations:** ^1^ Division of Gastroenterology and Hepatology, Mayo Clinic, Rochester, MN, United States; ^2^ Department of Surgery Mayo Clinic, Rochester, MN, United States

**Keywords:** metabolic and bariatric surgery, Roux-En-Y gastric bypass, sleeve gastrectomy, weight recurrence, transoral outlet reduction, endoscopic sleeve gastroplasty

## Abstract

Metabolic and bariatric surgery is the most effective therapy for weight loss and improving obesity-related comorbidities, comprising the Roux-en-Y gastric bypass (RYGB), gastric banding, sleeve gastrectomy (SG), and biliopancreatic diversion with duodenal switch. While the effectiveness of weight loss surgery is well-rooted in existing literature, weight recurrence (WR) following bariatric surgery is a concern. Endoscopic bariatric therapy presents an anatomy-preserving and minimally invasive option for managing WR in select cases. In this review article, we will highlight the endoscopic management techniques for WR for the most commonly performed bariatric surgeries in the United States –RYGB and SG. For each endoscopic technique, we will review weight loss outcomes in the short and mid-terms and discuss safety and known adverse events. While there are multiple endoscopic options to help address anatomical issues, patients should be managed in a multidisciplinary approach to address anatomical, nutritional, psychological, and social factors contributing to WR.

## 1. Introduction

**Figure 1:**
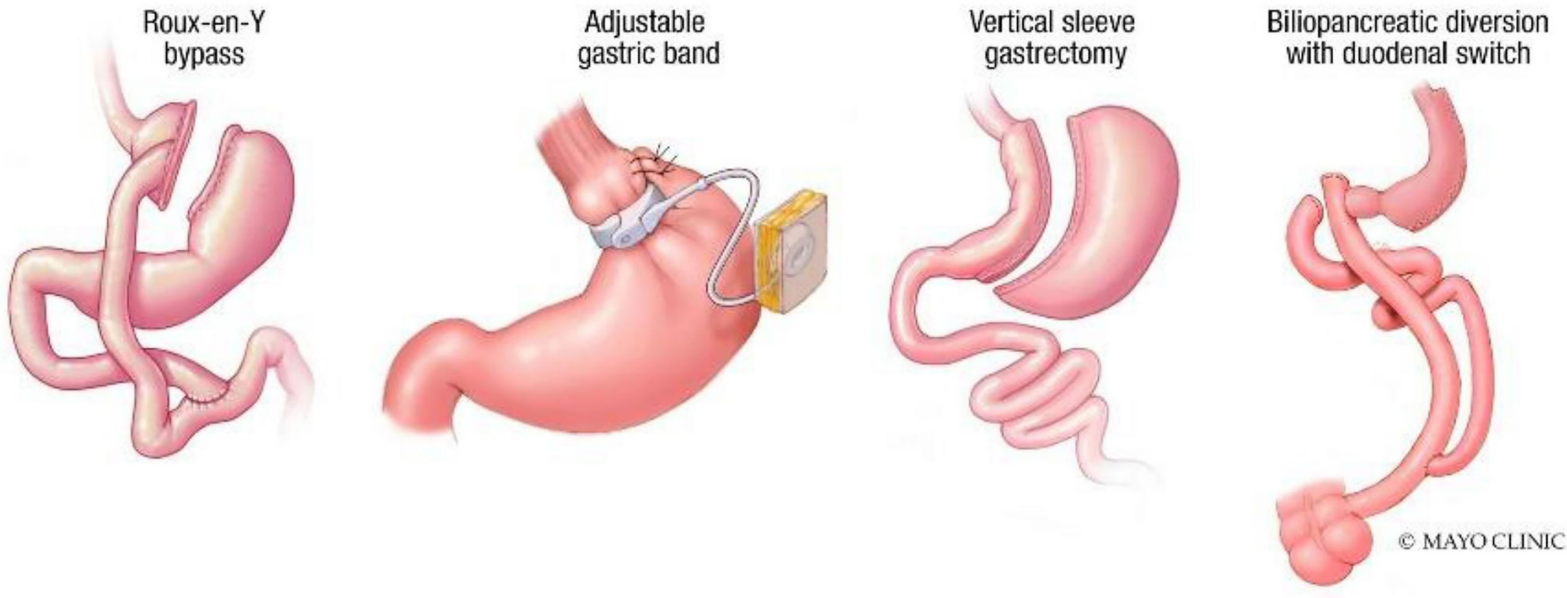
The different bariatric surgery modalities.

Bariatric surgery is the most effective therapy for weight loss and reducing obesity-related comorbidities, including diabetes, hypertension, sleep apnea, and nonalcoholic hepatic steatosis (NASH) (1). Metabolic and Bariatric Surgery (MBS) encompasses several procedures, including the Roux-en-Y gastric bypass (RYGB), gastric banding, sleeve gastrectomy (SG), and biliopancreatic diversion with duodenal switch (2) (**Figure 1**).

While the effectiveness of weight loss surgery is well-rooted in existing literature, weight recurrence (WR) following bariatric surgery is a concern. WR after MBS is defined as an increase of 25% excess weight loss (EWL) from nadir weight (3). This is also associated with the reappearance of weight-related comorbidities (4). After MBS, contributors to WR are multifactorial and include genetic, psychological, and behavioral determinants. Additionally, anatomic and technical factors specific to each bariatric surgery are associated with WR (4).

The management of post-procedural WR encompasses several approaches. Surgical revision and conversion to a different MBS, while necessary in some cases, are technically more challenging and associated with higher morbidity and mortality compared to the primary surgical intervention (5). Obesity pharmacotherapies are increasingly being utilized to manage WR though with variable efficacy, limited continuous accessibility, and consideration of cost and patient compliance (6). Endoscopic bariatric therapy presents an anatomy-preserving and minimally invasive option for managing WR in select cases. Several endoscopic revision options exist depending on the initial bariatric surgery type (7).

This review article will highlight endoscopic management options for WR after the most commonly performed bariatric surgeries in the United States – RYGB and SG. Notably, other less commonly performed bariatric surgery procedures, including the biliopancreatic diversion with duodenal switch (BPD/DS), are not associated with high rates of WR. Rather than only restricting the volume of food, this procedure significantly induces malabsorption, thereby rendering it physiologically more complex (but not impossible) to regain weight (8, 9).

For each endoscopic technique, we will review weight loss outcomes in the short and mid-terms and safety profile with a description of the known adverse effects.

## 2. Roux-En-Y Gastric Bypass

The RYGB (**Figure 1**) procedure creates a small 25 ml gastric pouch and separates it from the remaining (excluded) stomach. This pouch is connected to the jejunum, bypassing the more significant portion of the stomach. A separate distal bowel to bowel anastomosis (jejunojejunostomy) gives the RYGB its final configuration. The procedure is highly efficacious and results in approximately 35.8% total body weight loss (TBWL) at 1 year and 27.7% TBWL at 10 years (10).

Despite its efficacy, WR after RYGB is an escalating concern (11). Changes in anatomy, among other factors, have been shown to contribute to this pathology. Anatomically, the dilation of the gastrojejunal anastomosis (GJA), the dilation of the gastric pouch, and the presence of a gastrogastric fistula (GGF) are correlated with WR (12, 13).

Surgical revision of the GJA and gastric pouch for WR after RYGB is technically challenging due to the distorted surgical planes and anatomic changes from the index surgery (14). Revisional RYGB is at times associated with higher morbidity and increased length of hospital stay. While revisional surgery is effective and sometimes necessary to address WR and GGF (15, 16), it produces less weight loss than the primary operation (14). These challenges paved the way for the emergence and adoption of per-oral endoscopic approaches to address WR after RYGB. Since GJA dilation is correlated with WR, it rendered itself a potential WR management target. The introduction of full-thickness suturing and plication endoscopic platforms to reduce the GJA aperture and gastric pouch volume represented an important advancement in WR management after RYGB.

### 2.1. Transoral Outlet Reduction

**Figure 2:**
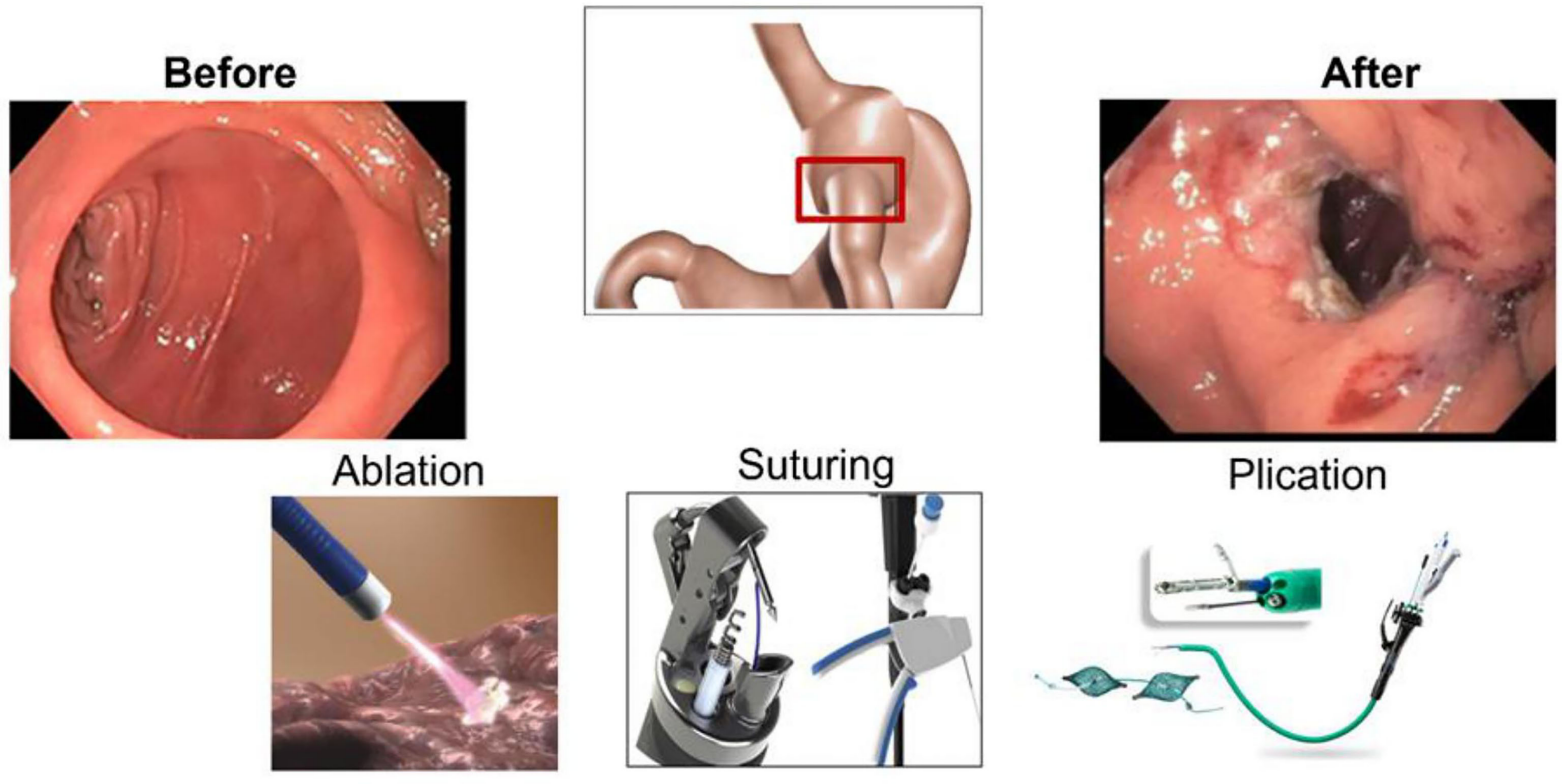
Available modalities for endoscopic management of weight regain after RYGB.

TORe is a therapeutic option for managing WR after RYGB by reducing the GJA diameter using commercially available endoscopic tools and platforms (**Figure 2**) (17). TORe reduces the size of the GJA to achieve weight loss through mechanical restriction; thus, decreasing hunger and improving satiety (18). There are several methods to perform TORe, including full-thickness endoscopic suturing, plications, and hybrid approaches with ablation or resection of the mucosal layer of the GJA (19, 20) (**Figure 2**).

#### 2.1.1. TORe With Endoscopic Suturing Platforms

TORe can easily reduce the GJA aperture using a commercially available endoscopic suturing device (17).

##### 2.1.1.1 Efficacy

A multicenter, randomized controlled trial studied an earlier version of the TORe procedure, utilizing a suction-based superficial suturing device (BARD Endocinch, Murray Hill, NJ). At 1-year follow-up, patients who underwent TORe experienced 3.5% TBWL, which was statistically significantly higher than those who underwent a sham procedure who experienced 0.4% TBWL (p=0.02) (21). Since then, the TORe approach has been further refined to enhance efficacy using full-thickness suturing and plication platforms, adjunct mucosal ablation before suturing, and evolution of suturing patterns and reinforcement in the gastric pouch (12, 22–25).

Argon plasma coagulation (APC) around the GJA has been utilized alone or with suturing to perform the TORe procedure. When serial APC sessions were compared with a single session TORe with APC around the GJA, both techniques were similarly efficacious with a comparable safety profile (26). APC-TORe has also been compared with a modified TORe with endoscopic submucosal dissection (ESD). At 12 months, combining ESD with suturing in TORe demonstrated more significant weight loss than APC-TORe ((12.1% ± 9.3% vs. 7.5% ± 3.3% TBWL) (p=0.04)) (27).

Similarly, TORe procedures have been enhanced by utilizing different suturing patterns, including the traditional interrupted suture pattern or the purse string method, with the latter being more effective pattern (22). Roughly similar %TBWL was reached with purse-string suturing in conjunction with mucosal ablation of the tissue at the GJA with either APC or ESD (28).

Finally, reinforcement suturing in the gastric pouch leads to pouch reduction, an example of which is the tubular TORe method. One study reported no significant difference in the %TBWL when pouch reduction was part of the TORe compared to standard TORe (29). However, Ghazi et al. demonstrated higher %TBWL at 9, 12 and 18 months utilizing tubular TORe with a U-shaped reinforced suturing sequence in the distal pouch compared to standard TORe (9.5% vs 4.6%, p=0.04 at 9 months; 7.7% vs 0.8%, p=0.009 at 12 months; 7.5% vs -2.1%, p=0.003 at 18 months) (30).

TORe has been studied with adjunctive pharmacotherapies, and with combined laparoscopic lengthening of the Roux-limb (31). These adjunct therapies enhanced efficacy and durability in the short and mid-term (12).

##### 2.1.1.2. Safety Profile

TORe is considered a minimally invasive and well-tolerated option for WR management after RYGB. One systematic review and meta-analysis demonstrated a pooled rate of adverse events (AEs) of 11.4%, with abdominal pain being the most common AE at 4.22%. The calculated pooled rate of mild, moderate, and severe AEs was 4.56%, 1.6%, and 0.57%, respectively (32). In another systemic review and meta-analysis of 130 patients, the overall reported post-procedure complications included nausea (13%), abdominal pain (17%), and a superficial esophageal tear that was successfully managed intraoperatively. No serious AEs were reported (17). In the RESTORe trial, the most common events occurred perioperatively or around the follow-up endoscopy, including nausea, vomiting, constipation, and pharyngolaryngeal pain (21). In a 5 -year retrospective study, the reported AEs rate of TORe was 3.9% (12). The AEs included submucosal esophageal tear due to overtube placement and bleeding at a suture site managed intraprocedural. These reports demonstrate a favorable benefit to risk ratio with the TORe procedure.

#### 2.1.2 TORe With Cryoablation Platforms

Cryoballoon ablation is a technique that utilizes cold nitrogen gas delivered *via* a catheter to freeze and induce superficial mucosal ablation. Cryoablation targeted at the gastric pouch and outlet is thought to cause fibrosis formation to achieve weight loss following WR from RYGB (33).

##### 2.1.2.1. Efficacy

In one retrospective chart review, cryoablation was performed in a subset of patients whose gastric pouch length exceeded 4 cm and had a gastric outlet size over 15 mm. Twenty-two patients were included in this study, with a resulting technical success rate of 89.5% for the gastric outlet and 93% for the pouch. TBWL was 8.1% (SD 12.8 %) at 8 weeks, and study investigators found a significant correlation between TBWL and outlet size reduction (coefficient – 0.28, *P* = 0.01) but not between TBWL and pouch size reduction (33).

##### 2.1.2.2. Safety Profile

Cryoablation has classically been used in the ablation of Barrett’s esophagus, with stricture formation being reported as an AE. When used for TORe, AEs were reported in three patients (13.6% of the study population, comprising two post-procedural cases of upper gastrointestinal bleeding secondary to gastric ulcer formation and one case of symptomatic outlet stenosis) (33).

#### 2.1.3. TORe With Argon Plasma Coagulation

APC utilizes a noncontact electrocoagulation method directing ionized gas to the tissue at the GJA, resulting in progressively reduced diameter (34). Using APC around the GJA was first demonstrated in 2006 as an adjunct to the standard TORe procedure. Increased weight loss was demonstrated in patients who underwent APC before suturing compared to those who had suturing alone (35).

APC is classically performed in several sessions, usually three sessions 6-8 weeks apart (36). The number of sessions varies depending on response, with discontinuation of treatment once the stoma reaches < 12 mm of breadth based upon an analysis showing diameters between 10-12 mm carrying the best risk-benefit ratio (37). Though still efficacious in producing weight loss, a smaller stoma diameter size is associated with a higher risk of obstructive symptoms (38). A dose-response relationship has also been observed with the electrocoagulation strength itself, as a higher-dose setting (70-80 watts) is associated with more significant weight loss than low dose APC of 45-55 watts (36).

##### 2.1.3.1. Efficacy

The efficacy of APC as a sole modality to perform TORe was demonstrated in multiple studies. A prospective, nonrandomized study evaluating serial APC performed in three sessions at intervals of 8 weeks showed a mean weight loss of 15.5 kg. However, the significant weight reduction did not surpass the initial weight loss following RYGB (38).

When APC was compared to a multidisciplinary lifestyle program with sham endoscopy in a randomized controlled trial, 15.6%TBWL (15.8 kg) was observed in the APC group compared to 9.3% TBWL (8.5 kg) in the control group at the end of 14 months of follow-up (p<0.001) (39). A multicenter retrospective chart review found differing weight loss outcomes based on initial post-RYGB BMI (body mass index). Mean TBWL peaked at the 24-month endpoint, 6.9% in the low BMI group, compared to 13.4% in the high BMI group (p< 0.0001) (40).

##### 2.1.3.2. Safety

The safety profile of APC is favorable. Stricture is often the most commonly seen AE. Large amounts of argon gas have been reported to cause abdominal discomfort and slight bowel distension and may occasionally induce a vagal response leading to hypotension and bradycardia (33).

### 2.2. Restorative Obesity Surgery, Endoluminal

ROSE is another procedure that uses full-thickness plications to manage WR after RYGB (34). It is a modified variant of the POSE: Primary Obesity Surgery Endoluminal procedure that uses the Incisionless Operating Platform (IOP; USGI, San Clemente, California). A tissue approximator, a tissue grasper, and a small caliber gastroscope are placed through the IOP to reduce the gastric pouch and anastomosis size. The tissue plication is performed by pulling the grasper into the approximator and aspirating the air to enlarge the plication surface. Then the needle deploys a pair of self-expanding tissue anchors, and the connecting suture is tightened (34).

#### 2.2.1. Efficacy

Multiple studies documented various weight loss outcomes of ROSE performed for WR after RYGB. One multicenter registry of 116 patients who underwent incisionless revision using the IOP to reduce the stoma and pouch size showed an average weight loss of 32% of WR from nadir at 6 months (n = 96) (41). In addition, one retrospective study analyzed the ROSE procedure’s outcome in 27 patients with WR following RYGB from 2008 to 2013. The %EWL was 9.3, 8.0, 6.7, -10.7, -13.5, - 5.8, -4.5 at6, 12, 24, 36, 48, 60 and 72 months, respectively. Although endoscopic plication achieved the expected reduction in the pouch and stoma diameter at 3 months, the patients regained the preoperative diameter at 12 months. This anatomical failure and the lack of follow-up may explain why most patients failed to achieve sustainable weight loss (42). A newer version of this procedure referred to as plication transoral outlet reduction (P-TORe), was studied in 111 patients. This procedure showed enhanced safety and efficiency. The %TBWL was 9.5% ± 8.5% at 12 months (p<0.0001). Predictors of weight loss were the amount of weight regain and baseline pouch length (43).

#### 2.2.2. Safety Profile

No severe AEs were described in the ROSE nor in the P-TORe procedure. Nonetheless, in the latter newer version, the overall AE rate was 12.6% including stenosis (9.9%), melena because of marginal ulceration (1.8%), and deep vein thrombosis (0.9%) (43).

## 3. Laparoscopic Sleeve Gastrectomy

LSG has rapidly gained popularity to become the most frequently performed bariatric surgery worldwide. According to the American Society for Metabolic and Bariatric Surgery’s latest report, LSG represented 59.4% of the 256,000 bariatric surgeries performed in 2019 (2). LSG entails longitudinally resecting the stomach on the greater curvature from the antrum starting opposite of the nerve of Latarjet up to the angle of His (44, 45).

LSG is highly effective at inducing weight loss and improving obesity-related comorbidities. However, WR occurs at rates of 5.7% at 2 years to 75.6% at 6 years (46). Anatomically, sleeve dilation has been reported as a basis for WR after LSG (47). One study showed that 30% of LSG patients require surgical revision for non-response, WR, and/or development of upper GI symptoms, mainly GERD (48). Surgical options for WR management after LSG are limited, with a conversion to a RYGB being the most commonly performed (49). One meta-analysis showed that the conversion from SG to RYGB resulted in desirable mid-term weight loss and potential resolution of GERD (50). However, some patients may have relative contraindications to RYGB, such as patients with a history of multiple complex abdominal operations, presence of bowel containing large hernia, active Crohn’s disease, previous bowel resections, and debilitating diarrhea (51, 52). These patients may benefit from having their sleeve anatomy restricted by per-oral endoscopic approaches.

### 3.1. Revisional Endoscopic Sleeve Gastroplasty

Abu Dayyeh et al. first described the feasibility of the ESG procedure as an alternative to SG in a single-center pilot study of 4 patients. This novel approach created a 2-row plication and reduced the entire stomach from the GE junction to the prepyloric antrum by making a small diameter sleeve (53).

#### 3.1.1. Efficacy

**Figure 3:**
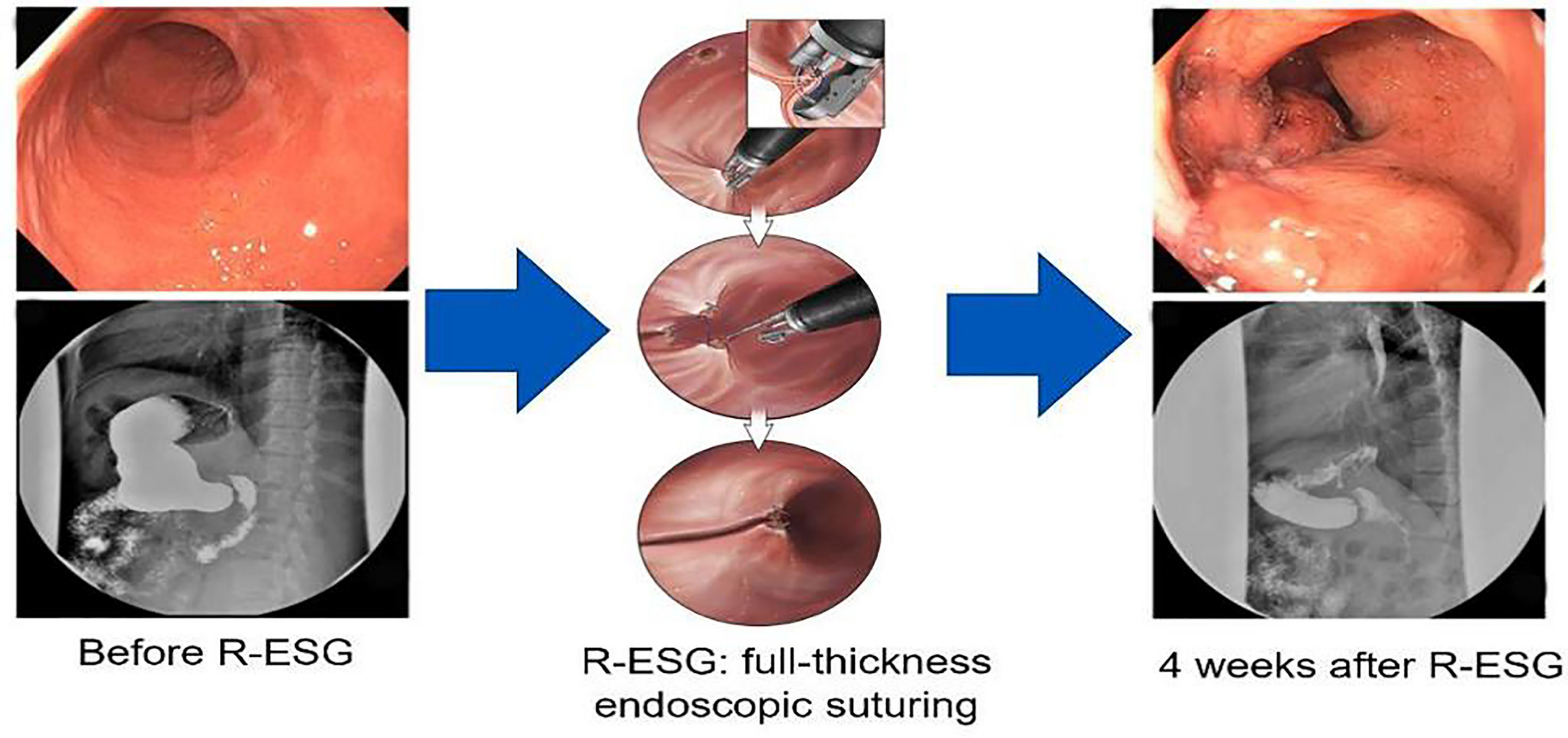
Endoscopic sleeve gastroplasty for endoscopic revision of sleeve gastrectomy.

Revisional ESG (**Figure 3**) after SG was first reported by Sharaiha et al. with a successful endoscopic sleeve plication after SG, which induced a weight loss of 9 kg (54). In a retrospective pilot case series of 5 patients who underwent revisional ESG for an enlarged gastric sleeve, a sustained TBWL of 6.7% to 17.2% was observed at 12 months (55). A later report described revisional ESG as a sleeve-in-sleeve procedure to create plications in the gastric body using a belt-and-suspenders pattern. The patient did well postprocedurally with reported weight loss at 3 months of 7 kg corresponding to 8% TBWL (56). In a multicenter retrospective study including 34 patients who underwent ESG for WR, 82.4% achieved ≥ 10% TBWL at 12 months. The median %TBWL was 13.2% and 18.3% at 6 months and 1 year, respectively (57). A more recent multicenter study included 82 patients who underwent revisional ESG for WR after LSG, showing ≥ 10% TBWL in 72.5% and 81% of patients at 6 months and 12 months. This study highlighted that revisional ESG is a safe and effective management option in the short-term (58).

#### 3.1.2. Safety

Revisional ESG has demonstrated a favorable safety profile with no reported serious AEs. Only one AE graded moderate in severity was observed due to the narrowing of the gastroesophageal junction, resulting in non-bloody emesis and resolved by endoscopic dilation (58). Hence, ESG presents a safe and minimally invasive alternative to surgery for WR after LSG.

## 4. Conclusion

WR following bariatric surgery is reported in a subset of patients and requires a multidisciplinary approach to management. When utilized as an adjunct to a comprehensive lifestyle and behavioral modification program, endoscopic revision is a minimally invasive and efficacious option. For patients who have undergone RYGB, standard or modified TORe has been the mainstay of endoscopic management. In patients with weight recidivism following SG, endoscopic sleeve gastroplasty is an evolving safe and effective approach in the short term. WR after bariatric surgery is multifactorial. While there are multiple endoscopic options to help address anatomical issues, patients should be managed in a multidisciplinary approach to address anatomical, nutritional, psychological, and social factors contributing to weight recidivism.

## Author Contributions

Manuscript concept and design: BA, DA, RY. Drafting of the manuscript: DA, RY, OG. Critical revision of the manuscript: OG, BA, DA, RY, BR, RG. All authors contributed to the article and approved the submitted version.

## Conflict of Interest

Author BA is a consultant for Endogenex, Endo-TAGSS, Metamodix, and BFKW; consultant and grant/research support from USGI, Cairn Diagnostics, Aspire Bariatrics, Boston Scientific; Speaker roles with Olympus, Johnson and Johnson; speaker and grant/research support from Medtronic, Endogastric solutions; and research support from Apollo Endosurgery, and Spatz Medica

The remaining authors declare that the research was conducted in the absence of any commercial or financial relationships that could be construed as a potential conflict of interest.

## Publisher’s Note

All claims expressed in this article are solely those of the authors and do not necessarily represent those of their affiliated organizations, or those of the publisher, the editors and the reviewers. Any product that may be evaluated in this article, or claim that may be made by its manufacturer, is not guaranteed or endorsed by the publisher.
